# Vegetarian Diet in Chronic Kidney Disease—A Friend or Foe

**DOI:** 10.3390/nu9040374

**Published:** 2017-04-10

**Authors:** Anna Gluba-Brzózka, Beata Franczyk, Jacek Rysz

**Affiliations:** 1 Department of Nephrology, Hypertension and Family Medicine, WAM Teaching Hospital of Lodz, Zeromskiego 113, 90-549 Lodz, Poland; 2 Department of Nephrology, Hypertension and Family Medicine, Medical University of Lodz, Zeromskiego 113, 90-549 Lodz, Poland; bfranczyk-skora@wp.pl (B.F.); jacek.rysz@umed.lodz.pl (J.R.)

**Keywords:** vegetarian diet, chronic kidney disease, malnutrition, glomerular filtration, benefits

## Abstract

Healthy diet is highly important, especially in patients with chronic kidney disease (CKD). Proper nutrition provides the energy to perform everyday activities, prevents infection, builds muscle, and helps to prevent kidney disease from getting worse. However, what does a proper diet mean for a CKD patient? Nutrition requirements differ depending on the level of kidney function and the presence of co-morbid conditions, including hypertension, diabetes, and cardiovascular disease. The diet of CKD patients should help to slow the rate of progression of kidney failure, reduce uremic toxicity, decrease proteinuria, maintain good nutritional status, and lower the risk of kidney disease-related secondary complications (cardiovascular disease, bone disease, and hypertension). It has been suggested that plant proteins may exert beneficial effects on blood pressure, proteinuria, and glomerular filtration rate, as well as results in milder renal tissue damage when compared to animal proteins. The National Kidney Foundation recommends vegetarianism, or part-time vegetarian diet as being beneficial to CKD patients. Their recommendations are supported by the results of studies demonstrating that a plant-based diet may hamper the development or progression of some complications of chronic kidney disease, such as heart disease, protein loss in urine, and the progression of kidney damage. However, there are sparse reports suggesting that a vegan diet is not appropriate for CKD patients and those undergoing dialysis due to the difficulty in consuming enough protein and in maintaining proper potassium and phosphorus levels. Therefore, this review will focus on the problem as to whether vegetarian diet and its modifications are suitable for chronic kidney disease patients.

## 1. Introduction

The main nutrition-related goals for people with chronic kidney disease (CKD) involve the slowing of kidney failure progression rate and minimizing uremic toxicity and metabolic disorders of kidney failure, diminishing proteinuria, maintaining good nutritional status, and lowering the risk of secondary complications including cardiovascular disease, bone disease, and disturbed blood pressure control [1]. However, nutrition requirements differ among patients with various stages of kidney function and with various comorbidities. The influence of diet on outcomes of patients with chronic kidney disease has been widely studied. Undernutrition has adverse effects on CKD patients’s prognosis, however, it remains unclear whether the correction of undernourishment can improve clinical outcomes in this group of patients. Also, the role of overnutrition is still not unraveled. According to studies, obesity is a risk factor for the development of kidney disease [2], but on the other hand, its presence is associated with better survival of CKD patients [3,4,5]. Overnutrition is associated with the development of hyperphosphatemia, hyperkalemia, sodium and volume overload, as well as the elevation in uremic metabolic products and their various potential deleterious effects [3,4,5]. The influence of most dietary interventions has not been widely examined in large randomized controlled trials in patients with CKD and therefore it is difficult to put forward any recommendations concerning proper nutritional goals and the most appropriate manner to achieve them in this vulnerable population.

## 2. Vegetarianism

For decades, vegetarianism and kidney disease have been thought to be impossible to combine. Plant-based diets, despite containing low amounts of protein, are also rich in potassium and phosphorus, and therefore they are believed to be unsuitable for CKD patients. However, evidence from clinical studies demonstrate that such a diet can be beneficial for CKD patients when they learn how to use it wisely. Nowadays, there are numerous types of vegetarianism, including lacto-ovo-vegetarianism, lacto-vegetarianism, ovo-vegetarianism, veganism, vitarianism, fruitarianism, liquidarianism, etc. Some of these diets combine plant-based diets with animal products such as eggs, milk, and honey, other are based on fruits or sprouts or juices. Regardless of the type of vegetarianism, such a diet typically contains large amounts of whole-grain cereals, nuts, fruits and vegetables, and is rich in dietary fibers, *n*-6 fatty acids, folic acid, potassium, magnesium, vitamin E, vitamin C, carotenoids, as well as many phytochemicals [6].

The results of small studies suggest that a plant-based diet can delay the progression of CKD, protect endothelium, help to control high blood pressure, and decrease proteinuria [7,8]. Such a diet provides not only nutrients, but it also protects against complications [9]. A plant-based diet stimulates butyrate-producing bacteria, which positively impact digestive tract health. These bacteria are beneficial for epithelial cells [10]. It is also a natural source of pre-biotics. Large bowel bacteria participating in the fermentation of fiber and protein contributes to the generation of short chain fatty acids (SCFAs). SCFAs promote cellular mechanisms maintaining colorectal tissue integrity [11,12,13], and while in circulation they influence immune function and inflammation [14]. Other beneficial bacteria (*Bifidobacterium*) generate vitamins, such as Folate, Biotin, K, Thiamine, and B12 [15]. Moreover, *Lactobacillus* and *Bifidobacterium* mediate the synthesis of bile acids and produce enzymes that are necessary for carbohydrate breakdown [16], and *Bacteroides thetaiotamicron* and *Faecalibacterium prausnitzii* generate anti-inflammatory compounds [17]. However, some products of fermentation, including phenols, ammonia, and hydrogen sulfide can also be toxic [13].

On the other hand, a vegetarian diet is relatively poor in long-chain *n*-3 fatty acids, zinc, iron, and vitamin B12. Some studies indicate that the risk of osteoporosis is higher in vegetarians than in the general population. Low intake of B12 vitamin is a problem in a vegetarian diet. Meat-eaters have much higher plasma vitamin B12 levels than vegetarians, but the cases of true vitamin B12 deficiency accompanied by hematological and neurological damage are very rare and have been reported only in vegans [18]. Bayesian meta-analysis of nine studies of 2749 subjects (1880 women and 869 men) assessing the effects of vegetarian diets on bone mineral density (BMD) in comparison to meat-eaters demonstrated that, despite the fact that BMD was lower in vegetarians and vegans, the results were not considered clinically significant [19]. Also, the European Prospective Investigation into Cancer and Nutrition EPIC-Oxford study failed to show significant differences in fracture rates between vegetarians and non-vegetarians [20].

Also, the low content of long-chain *n*-3 fatty acids, which are important for proper functioning of the cardiovascular system, seems to be a drawback of such a diet. However, the inclusion of eggs and dairy products should help to correct such a deficiency [21]. The content of iron in vegetarian and non-vegetarian diets is similar, however, significantly diminished iron absorption and iron bioavailability were observed in vegetarians. Surprisingly, hemoglobin and hematocrit levels remained within the normal range this group [22].

## 3. Protein-Energy Status

In patients with CKD, an appropriate amount of protein is required to avoid both malnutrition and overnutrition. Protein-energy malnutrition in CKD patients is a strong predictor of adverse outcomes. Its prevalence increases linearly with decreasing renal function in non-dialysis patients and may exceed 50% in those undergoing maintenance dialysis therapy [23]. The National Kidney Foundation (NKF) recommends a diet containing 0.6 g protein/kg/day for patients with advanced CKD (glomerular filtration rate <25 mL/min/1.73 m^2^) and 1.2 g protein/kg/day for patients with end-stage renal disease (glomerular filtration rate <10 mL/min/1.73 m^2^). In 2007, the National Kidney Foundation: K/DOQI Clinical Practice Guidelines and Clinical Practice Recommendations for Diabetes and Chronic Kidney Disease, recommended the limitation of dietary protein intake to 0.9 g/kg/day in patients with diabetes and CKD stage 2, as it was proved to be beneficial irrespective of established medical therapies [24]. The NKF’s 2013 guidelines suggest the reduction in protein intake to 0.8 g/kg/day in adults with diabetes (2C) or without diabetes (2B) and GFR <30 mL/min/1.73 m^2^ (glomerular filtration rate (GFR) categories G4–G5), with appropriate education and recommend the avoidance of high protein intake (41.3 g/kg/day) in adults with CKD at risk of progression (2C) [25]. In mild CKD, a low protein diet (≤0.6 g/kg/day) was not recommended by CARI (Caring for Australasians with Renal Impairment) guidelines due to the risk of malnutrition (1C) [26]. Caloric restriction is recommended only in case of overweight/obese patients with CKD. Such persons are encouraged to reduce their body mass index (BMI) to approach the range 18.5–24.9 kg/m^2^ and waist circumference ≤102 cm for men and ≤88 cm for women (2C). The summary of guidelines for patients with mild-moderate CKD is presented in Table 1.

Studies on animal models suggest that a vegetarian diet is suitable and nutritionally adequate in CKD [29,30,31]. Also, human studies confirm this thesis [32]. The large cohort EPIC-Oxford study assessed nutrient intakes in 33,863 meat-eaters and 31,546 non-meat-eaters [33]. This study revealed that mean energy intake was 5% lower in vegetarians and 14% lower in vegans in comparison to meat-eaters. Moreover, carbohydrate intake was higher in vegetarian individuals (51.2% vs. 46.9% of energy intake), while protein intake was lower (13.1% vs. 16.0% of energy intake) in comparison to meat-eaters. Although total fat intake was similar in these two groups, the content of polyunsaturated fat was substantially higher in vegetarians, while the percentage of saturated fat was significantly lower in this group [33]. The CKD study by Aparicio et al. [34] demonstrated that in regularly and carefully monitored CKD patients, vegetarian diets with a low or even a very low protein content supplemented with keto-analogues provided a satisfactory nutritional status. No considerable differences in body weight or triceps skinfold thickness were found in patients with diabetes with proteinuria after 8 weeks on a predominantly vegetable protein diet (0.7 g/kg), despite the fact that their previous consumption of total protein per day was higher [35]. In a study by Barsotti et al. [36], a transition from a traditional 1.0 g to 1.3 g protein/kg/day diet to a 0.7 g protein/kg/day vegan diet was not associated with substantial changes in serum total protein or albumin in patients with diabetes.

## 4. Impact on Glomerular Filtration Rate

Higher glomerular filtration rates have been demonstrated in patients with normal renal function on an animal-protein diet in comparison to persons on a vegetable-based diet [37,38]. Another study of patients on a high-protein diet (1 g/kg/day) of either soy, animal protein, or animal protein diet supplemented with fiber revealed significantly higher renal plasma flow, glomerular filtration rate, and proteinuria in those on the animal protein diet compared to individuals on the soy diet [8]. The transition from mixed animal–vegetable diet (1.0 to 1.3 g/kg/day) to a vegan diet (0.7 g/kg/day) was demonstrated to be associated with a significant decrease in glomerular filtration rate and proteinuria in patients with non-diabetic nephrosis [36]. Due to the change in the amount of consumed protein, it is difficult to assess whether the observed changes were associated with the modification of diet or with the reduced proteins load. However, the results of some studies suggest that the amount of protein is more important than its source in the progression of renal dysfunction. In a small study (eight patients with diabetes, proteinuria, and moderate renal insufficiency) the reduction in glomerular filtration rate was observed both in patients receiving a 1 g/kg animal protein diet and those on a 1 g/kg soy protein diet [37]. Similarly, Knight et al. [39] observed that an increase in protein consumption correlated with a decrease in glomerular filtration rate in women with mild renal insufficiency, irrespective of protein source.

## 5. Impact on Phosphorus Levels

In CKD patients, disturbances in mineral metabolism are frequent, and they begin usually at stage 3 and 4. As renal function decreases, the ability of kidneys to excrete phosphorus become more and more reduced. As a consequence, compensatory secondary hyperparathyroidism and elevations in fibroblast growth factor-23 (FGF23) occur in order to increase urinary phosphorus excretion and maintain phosphorus balance [40]. According to studies, hyperphosphatemia is an independent risk factor for mortality in dialysis patients and in patients with stages 3 and 4 CKD [41]. Therefore, phosphate consumption should be restricted in CKD patients. This tends to be difficult for patients on a typical Western diet, due to high phosphate content in dairy products and protein sources [42]. Moreover, in a Western diet, phosphorus amount is increased by the presence of preservatives added to processed and fast foods. According to estimates, the addition of polyphosphates for improving the appearance and shelf life of red and white meat may rise daily phosphorus loads by 300 to 500 mg [43]. Plant-based proteins are richer in phosphorus in comparison to animal proteins, however, the phosphate in plant proteins is only 30%–50% bioavailable, while animal proteins, for example, in milk and cheese, are estimated to be 70%–80% bioavailable. It is associated with high content of phytates in plant-based proteins. Phytates bind phosphorus and prevent its absorption [44]. Therefore, it seems that the consumption of grain-based vegetarian food may be associated with decreased phosphorus absorption compared with meat- or casein-based diets [42]. Animals studies confirm that rats with renal disorders fed with grain-based diet had lower serum phosphorus, urinary phosphorus excretion, and serum FGF23 levels in comparison to rats fed with standard synthetic casein animal diets [31]. Also, studies involving humans underline the importance of phosphate sources in general mineral metabolism. In a study by Moe et al. [42], patients on a vegetarian diet had lower serum phosphorus levels, a trend toward decreased urine 24-h phosphorus excretion, as well as significantly decreased FGF23 levels in comparison to patients on a meat-based diet, despite the fact that protein and phosphorus concentrations were equivalent in both diets. Also, in a crossover, randomized clinical trial involving patients with advanced diabetic nephropathy, a 65% vegetarian diet reduced albuminuria, urine urea nitrogen, and serum phosphorus levels in comparison to a 70% animal-based diet [45]. Therefore, it seems that a vegetarian diet may be beneficial for CKD patients, since it allows for increased protein consumption without increasing phosphorus levels above the required value. Further studies are required to unravel underlying mechanisms.

## 6. Impact on Insulin Resistance

Insulin resistance is a frequent disorder in patients with CKD. According to studies, it is closely associated with atherosclerosis [46,47], coronary heart disease (CHD) [48], incident CHD [49], incident stroke [50], and cardiovascular mortality in the general population [51,52] and it is considered to be an independent predictor of cardiovascular mortality in patients with various stages of CKD [53], as well as in those on maintenance dialysis [54]. Insulin resistance also plays an important role in the pathogenesis of protein-energy wasting in CKD patients [55]. According to studies, vegetarians seem to be more insulin sensitive than omnivores, and the degree of this sensitivity is associated with the period on vegetarian diet [56,57]. Other studies [8] reported the improvement in insulin sensitivity and reduction in fasting serum insulin levels, blood glucose levels, and endogenous glucose production in CKD patients on a vegetarian diet.

## 7. Impact on Metabolic Acidosis

In chronic kidney disease, dietary acid load may result in acidosis, despite a normal serum bicarbonate concentration [58]. Metabolic acidosis is quite a common complication observed in CKD patients, which stimulates endocrine, metabolic, and musculoskeletal abnormalities [32,59]. This state is aggravated by the consumption of large quantities of red meat and the consequent formation of fixed acids and acid-producing cationic amino acids. Vegetarian food contains plenty of organic bicarbonate-producing anions, including citrate, lactate, etc., and therefore it yields an alkaline load [60]. In the Dietary Approaches to Stop Hypertension (DASH) study, the comparison of a control diet with macronutrient and mineral content similar to average US consumption with a fruit and vegetable diet (sweets and grains were replaced with fruits and vegetables) revealed that the latter yielded considerably diminished dietary acid load (net endogenous acid production of 78 mEq/day vs. 31), despite comparable protein intake [61]. A study by Goraya et al. [62] reported that fruit and vegetable intake reduced net acid excretion by approximately one-third and was comparable to the administration of 0.5 mEq/kg/day of sodium bicarbonate. Moreover, the implementation of base-inducing fruits and vegetables into the diet has been demonstrated not only to reduce dietary acid load, but also to decrease proteinuria and blood pressure in patients with macroalbuminuric hypertensive nephropathy and stage 2 CKD, thus resulting in the hampering of CKD progression [62]. However, there is still a need for a large study with hard outcomes, assessing the safety and benefits of a foods-based approach to diminish dietary acid load in patients with early to moderate chronic kidney disease [63].

## 8. Cardiovascular Risk Factors

Advanced glycation end products (AGEs) and inflammatory cytokine concentrations are frequently elevated in patients with CKD [7,64]. This group of patients are susceptible to numerous inflammatory diseases, including cardiovascular disease, atherosclerosis, and stroke. A vegetarian diet is associated with cardiovascular benefits due to lower BMI, blood pressure, occurrence of hypertension, risk of type II diabetes (or improved glycemic control in diabetic and insulin-resistant individuals), and improved lipid profile in patients consuming plant-based food [65]. Animal foods are sources of saturated fat, while plant-based foods contain much less fat, and mainly monounsaturated and polyunsaturated fats. Fats contained in plants have cardioprotective properties, in contrast to that contained in animal foods. Animal products are lacking fibers and phytochemicals, which are known to be protective to vascular system [65]. While whole grain rice contains a lot of fibers, proteins, and no fat, an ounce of meat contains much more protein than plant food, no fibers, and plenty of saturated fats. Plant-based diets are rich not only in anti-inflammatory components, but also have a high capacity to neutralize free radicals (high oxygen radical absorbance capacity score; ORAC) [66].

The large EPIC-Oxford study demonstrated that age-adjusted mean BMI was highest in individuals on a meat-based diet, while the lowest values were observed in vegans [33]. Several studies reported the lowering of blood pressure values following the shift from a meat-based diet to a vegetarian diet [61,67]. Moreover, in a study by Mukkuden-Petersen et al. [68], the reduction in total cholesterol (TC) and low-density lipoprotein (LDL) cholesterol in vegetarians were observed in comparison to those on a meat diet. The switch from a meat to vegetarian diet also proved to be beneficial for normolipidemic and hyperlipidemic persons, since it decreased TC and LDL cholesterol levels. The correction of lipid profiles in vegetarians is associated with the high content of unsaturated fatty acids present in nuts, soy, and plant sterols [69]. There are numerous studies confirming cardiovascular benefits of a vegetarian diet. A 25% lower risk of cardiovascular disease (CAD) was observed in women who consumed nearly three servings of whole grains a day in comparison to those who ate less than one serving per week [70]. Whole grain consumption has been also demonstrated to reduce the risk of ischemic stroke, independently of known CAD risk factors (multivariate relative risk (RR) comparing the highest with the lowest quintile of intake: 0.69; 95% confidence interval (CI): 0.50, 0.98) [71]. A large Finnish study reported an inverse relationship between intake of vegetables and risk of both coronary artery disease and cardiovascular deaths [72]. In the multivariate analysis of RRs for CAD death, in which the highest and lowest tertiles of intake were compared, the following results were obtained: 0.66 (95% CI: 0.46–0.96; *p* = 0.02) for men and 0.66 (95% CI: 0.35–1.23; 0.35) for women (in case of vegetables) and 0.77 (95% CI: 0.52–1.12; *p* = 0.28) for men and 0.66 (95% CI: 0.36–1.22; *p* = 0.1) for women (in case of fruits) after the adjustment for age, smoking, serum cholesterol, hypertension, body mass index, and energy intake [72]. A meta-analysis of results of five prospective studies involving over 750,000 patients demonstrated 24% lower mortality from ischemic heart disease in vegetarians compared to non-vegetarians (death rate ratio: 0.76; 95% CI: 0.62, 0.94; *p* < 0.01) after over 10 years of follow-up [63]. The reduction in risk negatively correlated with the duration of the diet and compliance. However, in this study no association between a vegetarian diet and other major causes of death was found [63]. The abundance of nutrients and compounds exerting positive effects on cardiovascular system makes it impossible to determine the exact nutrients that are responsible for cardioprotection in a vegetarian diet.

## 9. Other Effects

Soy consumption has been demonstrated to be beneficial in patients with chronic kidney disease, due to cardiovascular protection, decreased proteinuria, and cancer prevention [7,73]. A meta-analysis of nine trials involving 197 subjects demonstrated that the intake of soy protein significantly reduced serum creatinine (mean difference −6.231 μmol/L; 95% Cl: −11.109, −1.352 μmol/L; *p* = 0.012) and serum phosphorus concentrations (−0.804; 95% CI: −1.143, −0.464 μmol/L; *p* = 0.00) [74]. Moreover, it considerably reduced serum triglycerides (TG) (−0.223 mmol/L; 95% CI: −0.396, −0.051 mmol/L; *p* = 0.011) without affecting TC or Ca levels.

In vegetarian patients undergoing hemodiafiltration (HDF), the levels of indoxyl sulfate (IS) and p cresyl sulfate (PCS) (protein bound toxins that accumulate in CKD) were also demonstrated to be lower [75]. The authors suggested that a vegetarian diet might potentially reduce IS and PCS production by the intestinal microbiome. Moreover, they observed lower pre-on-line HDF serum urea and phosphate (13.8 ± 3.8 vs. 18.4 ± 5.2 mmol/L, and 1.33 ± 0.21 vs. 1.58 ± 0.45 mmol/L; *p* < 0.05) as well as estimated urea nitrogen intake (1.25 ± 0.28 vs. 1.62 ± 0.5 g/kg/day; *p* < 0.05), respectively.

The effect of a vegetarian diet in CKD patients has been examined in a few clinical trials. Specialized vegetarian diet in patients with 3 and 4 stage CKD was associated with the maintenance or increase in body weight, serum total protein, serum albumin, and transferrin in comparison to patients on a conventional low-protein diet [36]. The comparison of vegetarian hemodialysis patients with non-vegetarian ones did not reveal any significant differences in serum albumin or prealbumin. However, serum phosphorus and parathyroid hormone were found to be significantly lower in the vegetarian group [76]. In a study in which hemodialysis patients were first on a vegetarian diet for a week and then, after 2–4 weeks of washout, were switched to a meat diet, significantly lower plasma phosphorus was noted in those on the plant-based diet (mean (standard deviation, SD) serum phosphorus 3.7 (0.6) after a meat-based diet vs. 3.2 (0.5) after a vegetarian diet, *p* = 0.02) [42]. Moreover, Moe et al. [42] revealed significantly lower FGF23 (which is a sensitive marker of phosphorus metabolism changes) in HD patients on a vegetarian diet.

## 10. The Safety of Vegetarian Diet

A vegetarian diet was also suggested to be a safe option for pregnant patients with CKD. Due to the fact that pregnancy induces hyperfiltration, diets with restricted amount of protein should be beneficial in this group of patients [77]. In the study by Piccoli et al. [77], pregnant patients with severe kidney impairment and/or nephrotic proteinuria were placed on a vegetarian diet in which protein amount was compromised between the goal of diminishing hyperfiltration and increased metabolic needs of pregnancy. Although in their study no beneficial effects of diet were observed, there were also no negative effects of vegetarian diet assessed on the basis of baby health outcomes. A supplemented vegetarian low-protein diet (0.6–0.7 g/kg per day) turned out to be sufficient for the maintenance of satisfactory nutritional status during the pregnancy and after delivery, even in breast-feeding women. Moreover, most of the children of the vegetarian women had normal intrauterine growth and developed normally between 1 month and 7.5 years from delivery [77]. The review of the results obtained during 15 years of treatment of pregnant CKD women on moderately restricted low-protein diets confirms that such a diet is a safe option in the management of pregnant CKD patients [78]. A trend towards better preserved fetal growth was observed. Results of animal studies suggest that the beneficial effects of vegetable proteins and supplementation with ketoacids is associated with endothelium protective properties of ketoacids in rats with kidney disease and a decrease in the risk of CKD in the offspring of rats with genetic kidney diseases that are fed a soy rich diet [79,80].

Vegetarian diet supplemented with a very-low-protein diet was also demonstrated to be safe for pre-dialysis patients, since it exerted no detrimental effect on the short- and long-term outcomes of patients, even those already on renal replacement therapy [81]. A prospective, randomized, controlled trial of the safety and efficacy of a ketoanalogue-supplemented vegetarian very low-protein diet (KD; 0.3 g/kg vegetable proteins and 1 cps/5 kg ketoanalogues per day) revealed the correction of metabolic abnormalities, no changes in nutritional parameters, and no adverse reactions in non-diabetic adults with stable Egfr <30 mL/min per 1.73 m^2^. Therefore, the authors stated that a KD diet was nutritionally safe and could postpone dialysis initiation in some patients with CKD [82]. In dialysis patients, a vegetarian diet was associated with lower serum blood urea nitrogen, creatinine, muscle mass, BMI, and inflammatory marker levels, however, they required a higher erythropoietin dose. The comparison of albumin, prealbumin, muscle strength, subjective global assessment, and activities of daily living between vegetarian HD patients and non-vegetarian HD patients revealed no differences between these two groups [76]. The results of studies suggest that a vegetarian diet, even when combined with low protein intake, appears to be suitable for CKD patients and to provide adequate nutrition; however, it should be implemented following appropriate planning and consultation with dietician. Patients with potassium concerns should avoid potassium-rich plant proteins, such as seitan or tofu, and replace them with nuts or soy beans.

## 11. Conclusions

On the basis of the presented results, it seems that a vegetarian diet provides adequate nutrition and exerts numerous beneficial effects. The impact of a vegetarian diet is summarized in Table 2. A well-planned vegetarian diet is associated with cardiovascular benefits and the correction of CKD-accompanying complications. Its benefits are associated with the large amounts of dietary fibers, n-6 fatty acids, folic acid, potassium, magnesium, vitamin E, vitamin C, and carotenoids, as well as the many phytochemicals contained. Moreover, patients who decide to switch to a vegetarian diet may also adopt other good lifestyle practices, including higher levels of physical activity, refraining from smoking, and decreased consumption of alcohol. Due to the fact that a vegetarian food contains less proteins in comparison to a meat-based diet, it seems to be good for CKD patients. However, reports concerning the influence of a vegetarian diet on the progression of renal failure are still conflicting. According to some studies, the consumption of a vegetarian diet slows down the progression of renal injury, while others indicated that high protein intake is likely to accelerate CKD progression independently of the source of proteins (plant or animal). Therefore, a vegetarian diet may be beneficial due to the associated cardioprotective, anti-oxidant, and lipid-lowering properties, however, it has to meet protein requirements to provide adequate nutrition in CKD patients. Due to the fact that some of the beneficial effects of a vegetarian/vegan diet may also be confounded by a healthier lifestyle, the adoption of a vegetarian/vegan diet seems to be an option for some CKD patients.

## Figures and Tables

**Table 1:** Guidelines for patients with mild to moderate chronic kidney disease (CKD).

Guideline	Protein Intake	Salt Restriction	Phosphorus	Serum Potassium	Acid-Base Balance	Reference
KDIGO 2012	0.8 g/kg/day in adults with diabetes (2C) or without diabetes (2B) and GFR <30 mL/min/1.73 m^2^ (GFR categories G4–G5)	<90 mmol (<2 g) per day of sodium (5 g of sodium chloride) in adults	-	-	-	[25]
KHA-CARI (2013) Mild CKD (stages 1–3)	0.75–1.0 g/kg/day	100 mmol/day (2.3 g sodium or 6 g salt per day) or less	No restriction of dietary phosphate intake	Restriction by appropriately suited diet	-	[26]
ESPEN (2000) Early to moderate renal failure	0.55–0.6 (2/3 of HBV) (minimum protein requirement)	-	Dietary phosphorus intake 600–700 mg/day	-	-	[27]
UK Renal Association (2009–2010)	0.75 g/kg IBW/day for patients with stage 4–5 CKD not on dialysis 1.2 g/kg IBW/day for patients treated with dialysis (2B)	-	-	-	-	[28]

HBV—high biological value; IBW—Ideal Body Weight; ANAES—French National Agency for Accreditation and Evaluation in Health (Agence Nationale d’Accréditation et d’ Evaluation en Santé); KHA-CARI—Kidney Health Australia-Caring for Australasians with Renal Impairment; ESPEN—European Society of Parenteral and Enteral Nutrition (ESPEN).

**Table 2:** The summary of results of described studies.

Country	Diet/Groups	Time	Outcome	Reference
UK/Italy	Healthy individuals fed with vegetable protein diet (*n* = 10), an animal protein diet (*n* = 10), or an animal protein diet supplemented with fiber (*n* = 7). All diets contained the same amount of total protein.	3 weeks	GFR, renal plasma flow, and fractional clearance of albumin and IgG were significantly higher on the animal than the vegetable protein diets (GFR: 121 ± 4 vs. 111 ± 4 mL/min/1.73 m^2^, *p* < 0.001; RPF: 634 ± 29 vs. 559 ± 26 mL/min/1.73 m^2^, *p* < 0.001; theta Alb: (19.5 ± 3.1) × 10^−7^ vs. (10.2 ± 1.6) × 10^−7^, *p* < 0.01; theta IgG: (11.6 ± 3.1) × 10^−7^ vs. (7.5 ± 1.7) × 10^−7^, *p* < 0.05).	[8]
USA	1624 women with normal renal function or mild renal insufficiency enrolled in the Nurses’ Health Study. High protein diet	11 years	High protein intake was not significantly associated with change in estimated GFR in women with normal renal function. In women with mild renal insufficiency, protein intake was significantly associated with a change in estimated GFR of −1.69 mL/min per 1.73 m^2^ (CI, −2.93 to −0.45 mL/min per 1.73 m^2^) per 10 g increase in protein intake. High intake of non-dairy animal protein in women with mild renal insufficiency was associated with a significantly greater change in estimated GFR (−1.21 mL/min per 1.73 m^2^ (CI, −2.34 to −0.33 mL/min per 1.73 m^2^)	[39]
USA	Nine patients with a mean estimated GFR of 32 mL/min. Comparison of vegetarian and meat diets	1 week	1 week of a vegetarian diet led to lower serum phosphorus levels and decreased FGF23 levels. Plasma phosphorus (mg/dL) before meat consumption 3.5 ± 0.6 and after it 3.7 ± 0.6; plasma phosphorus (mg/dL) before vegetarian diet 3.5 ± 0.6 and after it 3.2 ± 0.5; *p* = 0.02. Plasma intact PTH (pg/mL) before meat 58 ± 31, after meat 46 ± 29, before vegetarian diet 58 ± 39, and after it 56 ± 30, *p* = 0.002. Plasma FGF23 (pg/mL) before meat 72 ± 39 and after it 101 ± 83; before vegetarian diet 84 ± 65 and after it 61 ± 35, *p* = 0.008.	[42]
Isfahan, Iran	14 patients (10 men and 4 women). Group 1—0.8 g/kg protein (70% animal and 30% vegetable proteins) Group 2—diet with the same amount of protein with 35% animal protein, 35% soy protein, and 30% other vegetable proteins.	7 weeks	Consumption of soy protein reduced urinary urea nitrogen (−0.9 ± 0.8 vs. 0.2 ± 0.6 mg/dL, respectively, SD; *p* < 0.001), proteinuria (−78 ± 37 vs. 42 ± 39 mg/day, respectively, SD; *p* < 0.001), blood sodium (−2 ± 0.04 vs. 2.0 ± 0.06 mg/dL, respectively, SD; *p* < 0.01), and serum phosphorus (−0.03 ± 0.2 vs. 0.2 ± 0.3 mg/dL, respectively, SD; *p* < 0.01) compared with animal protein	[45]
Hualien, Taiwan	98 healthy female adults: 49 Buddhist lactovegetarians and 49 omnivores.	-	Vegetarians had significantly lower levels of fasting insulin (median: 35.3 vs. 50.6 pmol/L) and plasma glucose (mean: 4.7 (se 0.05) vs. 4.9 (se 0.05) mmol/L) in comparison to omnivores. Insulin resistance (homeostasis model assessment method) was significantly lower in the vegetarians than in the omnivores (median: 1.10 vs. 1.56)	[56]
Chia-Yi, Taiwan	36 healthy volunteers (vegetarian, *n* = 19; omnivore, *n* = 17) with normal fasting plasma glucose levels	-	Omnivores had higher serum uric acid levels than vegetarians (5.25 ± 0.84 vs. 4.54 ± 0.75 mg/dL, *p* = 0.011). Significant differences between omnivores and vegetarians: fasting insulin, 4.06 ± 0.77 vs. 3.02 ± 1.19 microU/mL, *p* = 0.004; HOMA-IR, 6.75 ± 1.31 vs. 4.78 ± 2.07, *p* = 0.002; HOMA %S, 159.2% ± 31.7% vs. 264.3% ± 171.7%, *p* = 0.018)	[57]
Oxford, UK	37,875 healthy men and women participating in EPIC-Oxford. Four diet groups (meat-eaters, fish-eaters, vegetarians, and vegans)	-	Age-adjusted mean BMI was significantly different between the four diet groups, being highest in the meat-eaters (24.41 kg/m^2^ in men, 23.52 kg/m^2^ in women) and lowest in the vegans (22.49 kg/m^2^ in men, 21.98 kg/m^2^ in women)	[33]
Toronto, Ontario, Canada	Volunteers who were already consuming a low-saturated fat, low-cholesterol diet before starting the study. Test (combination) diet—very low content of saturated fat and high in plant sterols (1 g/1000 kcal), soy protein (23 g/1000 kcal), and viscous fibers (9 g/1000 kcal) obtained from foods available in supermarkets and health food stores	1 month	The diet reduced low-density lipoprotein (LDL)-cholesterol by 29.0% ± 2.7% (*p* < 0.001) and the ratio of LDL-cholesterol to high-density lipoprotein (HDL)-cholesterol by 26.5% ± 3.4% (*p* < 0.001). Maximal reductions were seen by week 2.	[69]
Boston, MA, USA	75,521 women aged 38–63 year with no previous history of cardiovascular disease or diabetes. Assessment of whole-grain consumption effects	10 years. of follow-up	After adjustment for age and smoking, increased whole-grain intake was associated with decreased risk of CHD. For increasing quintiles of intake, the corresponding relative risks (RRs) were 1.0 (reference), 0.86, 0.82, 0.72, and 0.67 (95% CI comparing two extreme quintiles: 0.54, 0.84; *p* for trend <0.001).	[70]
-	Meta-analysis of 12 studies (280 participants). Assessment of the effects of soy protein containing isoflavones in patients with chronic kidney disease	NA ^1^	Dietary soy was associated with significant decrease of serum creatinine (−0.05 mg/dL (95% CI: −0.10, −0.00 mg/dL; *p* = 0.04)), serum phosphorus (−0.13 mg/dL (95% CI: −0.26, −0.01 mg/dL; *p* = 0.04)), CRP (−0.98 mg/L (95% CI: −1.25, −0.71 mg/L; *p* < 0.00001)), and proteinuria (−0.13 mg/day (95% CI: −0.18, −0.08 mg/day; *p* < 0.00001)) in the pre-dialysis subgroup.	[73]
-	The meta-analysis consisted of nine trials, comprising 197 subjects. Analysis of effects of soy protein on chronic kidney disease	NA	Soy protein intake significantly reduced SCR (−6.231 μmol/L (95% confidence interval (CI): −11.109, −1.352 μmol/L); *p* = 0.012) and serum phosphorus concentrations (−0.804 (95% CI: −1.143, −0.464 μmol/L); *p* = 0.00). It also significantly reduced serum TG, with a pooled estimated change of −0.223 mmol/L (95% CI: −0.396, −0.051 mmol/L; *p* = 0.011).	[74]
London, UK; Brighton, UK; Ash Sharqia Governorate, Egypt	Cohort of 138 CKD stage 5 patients treated by On-line HDF (Ol-HDF). Comparison of indoxyl sulfate (IS) and *p*-cresyl sulfate (PCS) in vegetarians and omnivores.	-	Vegetarian patients had lower IS and PCS levels (median 41.5 (24.2–71.9) vs. 78.1 (49.5–107.5) and PCS (41.6 (14.2–178.3) vs. 127.3 (77.4–205.6) µmol/L, respectively, *p* < 0.05, as well as lower pre-Ol-HDF serum urea, and phosphate (13.8 ± 3.8 vs. 18.4 ± 5.2 mmol/L, and 1.33 ± 0.21 vs. 1.58 ± 0.45 mmol/L), and estimated urea nitrogen intake (1.25 ± 0.28 vs. 1.62 ± 0.5 g/kg/day), respectively, all *p* < 0.05	[75]

^1^ NA: does not apply.

## References

[1] Renal Dietitians Dietetic Practice Group, National Kidney Foundation Council on Renal Nutrition (2002). National Renal Diet: Professional Guide.

[2] Hsu C.Y., McCulloch C., Iribarren C., Darbinian J., Go A. (2006). Body mass index and risk for end-stage renal disease. Ann. Intern. Med..

[3] Kovesdy C.P., Kopple J.D., Kalantar-Zadeh K. (2013). Management of protein-energy wasting in non-dialysis-dependent chronic kidney disease: Reconciling low protein intake with nutritional therapy. Am. J. Clin. Nutr..

[4] Kalantar-Zadeh K., Abbott K.C., Salahudeen A.K., Kilpatrick R.D., Horwich T.B. (2005). Survival advantages of obesity in dialysis patients. Am. J. Clin. Nutr..

[5] Kovesdy C.P., Anderson J.E., Kalantar-Zadeh K. (2007). Paradoxical association between body mass index and mortality in men with CKD not yet on dialysis. Am. J. Kidney Dis..

[6] Davey G.K., Spencer E.A., Appleby P.N., Allen N.E., Knox K.H., Key T.J. (2003). EPIC-Oxford: Lifestyle characteristics and nutrient intakes in a cohort of 33,883 meat-eaters and 31,546 non meat-eaters in the UK. Public Health Nutr..

[7] Azadbakht L., Atabak S., Esmaillzadek A. (2008). Soy protein intake, cardiorenal indices, and C reactive protein in type II diabetes with nephropathy; a longitudinal randomized clinical trial. Diabetes Care.

[8] Kontessis P., Jones S., Dodds R., Trevisan R., Nosadini R., Fioretto P., Borsato M., Sacerdoti D., Viberti G. (1990). Renal, metabolic and hormonal responses to ingestions of animal and vegetable proteins. Kidney Int..

[9] Elliott P., Stamler J., Dyer A.R., Appel L., Dennis B., Kesteloot H., Ueshima H., Okayama A., Chan Q., Garside D.B. (2006). Association between Protein intake and blood pressure. Arch. Intern. Med..

[10] Barcenilla A., Pryde S.E., Martin J.C., Duncan S.H., Stewart C.S., Henderson C., Flint H.J. (2000). Phylogenetic relationships of butyrate-producing bacteria from the human gut. Appl. Environ. Microbiol..

[11] Cummings J.H., Macfarlane G.T. (1991). The control and consequences of bacterial fermentation in the human colon. J. Appl. Bacteriol..

[12] Donohoe D.R., Garge N., Zhang X., Sun W., O’Connell T.M., Bunger M.K., Bultman S.J. (2011). The microbiome and butyrate regulate energy metabolism and autophagy in the mammalian colon. Cell Metab..

[13] Conlon M.A., Bird A.R. (2015). The Impact of Diet and Lifestyle on Gut Microbiota and Human Health. Nutrients.

[14] Trent M.S., Stead C.M., Tran A.X., Hankins J.V. (2006). Diversity of endotoxin and its impact on pathogenesis. J. Endotoxin Res..

[15] Nicholson J.K., Holmes E., Kinross J., Burcelin R., Gibson G., Jia W., Pettersson S. (2012). Host-gut microbiota metabolic interactions. Science.

[16] Xu J., Bjursell M.K., Himrod J., Deng S., Carmichael L.K., Chiang H.C., Hooper L.V., Gordon J.I. (2003). A genomic view of the human-Bacteroides thetaiotaomicron symbiosis. Science.

[17] Sokol H., Pigneur B., Watterlot L., Lakhdari O., Bermudez-Humaran L.G., Gratadoux J.-J., Blugeon S., Bridonneau C., Furet J.-P., Corthier G. (2008). Faecalibacterium prausnitzii is an anti-inflammatory commensal bacterium identified by gut microbiota analysis of Crohn disease patients. Proc. Natl. Acad. Sci. USA.

[18] Herrmann W., Geisel J. (2002). Vegetarian lifestyle and monitoring of vitamin B-12 status. Clin. Chim. Acta.

[19] Ho-Pham L.T., Nguyen N.D., Nguyen T.V. (2009). Effect of vegetarian diets on bone mineral density: A Bayesian meta-analysis. Am. J. Clin. Nutr..

[20] Appleby P.N., Roddam A., Allen N., Key T.J. (2007). Comparative fracture risk in vegetarians and nonvegetarians in EPIC-Oxford. Eur. J. Clin. Nutr..

[21] Craig W.J. (2009). Health effects of vegan diets. Am. J. Clin. Nutr..

[22] Rizzo N.S., Jaceldo-Siegl K., Sabate J., Fraser G.E. (2013). Nutrient Profiles of Vegetarian and Non-vegetarian Dietary Patterns. J. Acad. Nutr. Diet..

[23] NKF KDOQI GUIDELINES 2002. KDOQI Clinical Practice Guidelines for Chronic Kidney Disease: Evaluation, Classification, and Stratification. http://www2.kidney.org/professionals/kdoqi/guidelines_ckd/p6_comp_g9.htm.

[24] National Kidney Foundation (2007). K/DOQI Clinical Practice Guidelines and Clinical Practice Recommendations for Diabetes and Chronic Kidney Disease. Am. J. Kidney Dis..

[25] National Kidney Foundation KDIGO 2012 Clinical Practice Guideline for the Evaluation and Management of Chronic Kidney Disease. www.kdigo.org/clinical_practice_guidelines/pdf/CKD/KDIGO_2012_CKD_GL.pdf.

[26] Kidney Health Australia—Caring for Australasians with Renal Impairment (KHA-CARI) Guidelines Modification of Lifestyle and Nutrition Interventions for Management of Early Chronic Kidney Disease. http://www.cari.org.au/CKD/CKD%20early/Modification_of_Llifestyle_Nutrition_ECKD.pdf.

[27] Toigo G., Aparicio M., Attman P.O., Cano N., Cianciaruso B., Engel B., Fouque D., Heidland A., Teplan V., Wanner C. (2000). Consensus Report. Expert Working Group report on nutrition in adult patients with renal insufficiency. Clin. Nutr..

[28] Clinical Practice Guidelines (2009–2010). Nutrition in CKD. UK Renal Association. 5th Edition. www.renal.org/guidelines.

[29] Ogborn M.R., Bankovic-Calic N., Shoesmith C., Buist R., Peeling J. (1998). Soy protein modification of rat polycystic kidney disease. Am. J. Physiol..

[30] Trujillo J., Ramirez V., Perez J., Torre-Villalvazo I., Torres N., Tovar A.R., Munoz R.M., Uribe N., Gamba G., Bobadilla N.A. (2005). Renal protection by a soy diet in obese Zucker rats is associated with restoration of nitric oxide generation. Am. J. Physiol. Ren. Physiol..

[31] Moe S.M., Chen N.X., Seifert M.F., Sinders R.M., Duan D., Chen X., Liang Y., Radcliff J.S., White K.E., Gattone V.H. (2009). A rat model of chronic kidney disease-mineral bone disorder. Kidney Int..

[32] Mitch W.E., Remuzzi G. (2016). Diets for patients with chronic kidney disease, should we reconsider?. BMC Nephrol..

[33] Spencer E.A., Appleby P.N., Davey G.K., Key T.J. (2003). Diet and body mass index in 38,000 EPIC-Oxford meat-eaters, fish-eaters, vegetarians and vegans. Int. J. Obes. Relat. Metab. Disord..

[34] Aparicio M., Chauveau P., de Précigout V., Bouchet J.L., Lasseur C., Combe C. (2000). Nutrition and outcome on renal replacement therapy of patients with chronic renal failure treated with supplemented very low-protein diet. J. Am. Soc. Nephrol..

[35] Jibani M.M., Bloodworth L.L., Foden E., Griffiths K.D., Galpin O.P. (1991). Predominantly vegetarian diet in patients with incipient and early clinical diabetic nephropathy: Effects of albumin excretion rate and nutritional status. Diabet. Med..

[36] Barsotti G., Morelli E., Cupisti A., Meola M., Dani L., Giovannetti S. (1996). A low-nitrogen low-phosphorous vegan diet for patients with chronic renal failure. Nephron.

[37] Anderson J.W., Blake J.E., Turner J., Smith B.M. (1998). Effects of soy protein on renal function and proteinuria in patients with type 2 diabetes. Am. J. Clin. Nutr..

[38] Lohsiriwat S. (2013). Protein Diet and Estimated Glomerular Filtration Rate. Open J. Nephrol..

[39] Knight E.L., Stampfer M.J., Hankinson S.E. (2003). The impact of protein intake on renal function decline in women with normal renal function or mild renal insufficiency. Ann. Intern. Med..

[40] Nagano N., Miyata S., Abe M., Kobayashi N., Wakita S., Yamashita T., Wada M. (2006). Effect of manipulating serum phosphorus with phosphate binder on circulating PTH and FGF23 in renal failure rats. Kidney Int..

[41] Kestenbaum B., Sampson J.N., Rudser K.D., Patterson D.J., Seliger S.L., Young B., Sherrard D.J., Andress D.L. (2005). Serum phosphate levels and mortality risk among people with chronic kidney disease. J. Am. Soc. Nephrol..

[42] Moe S.M., Zidehsarai M.P., Chambers M.A., Jackman L.A., Radcliffe J.S., Trevino L.L., Donahue S.E., Asplin J.R. (2011). Vegetarian compared with meat dietary protein source and phosphorus homeostasis in chronic kidney disease. Clin. J. Am. Soc. Nephrol..

[43] Sullivan C., Sayre S.S., Leon J.B. (2009). Effect of food additives on hyperphosphatemia among patients with end-stage renal disease. A randomized controlled trial. J. Am. Med. Assoc..

[44] Pagenkemper J. (1995). Planning a vegetarian renal diet. J. Ren. Nutr..

[45] Azadbakht L., Esmaillzadeh A. (2009). Soy-protein consumption and kidney-related biomarkers among type 2 diabetics: A crossover, randomized clinical trial. J. Ren. Nutr..

[46] Howard G., O’Leary D.H., Zaccaro D., Haffner S., Rewers M., Hamman R., Selby J.V., Saad M.F., Savage P., Bergman R. (1996). Insulin sensitivity and atherosclerosis. The Insulin Resistance Atherosclerosis Study (IRAS) Investigators. Circulation.

[47] Folsom A.R., Eckfeldt J.H., Weitzman S., Ma J., Chambless L.E., Barnes R.W., Cram K.B., Hutchinson R.G. (1994). Relation of carotid artery wall thickness to diabetes mellitus, fasting glucose and insulin, body size, and physical activity. Atherosclerosis Risk in Communities (ARIC) Study Investigators. Stroke.

[48] Kahn S.E., Leonetti D.L., Prigeon R.L., Boyko E.J., Bergstrom R.W., Fujimoto W.Y. (1995). Relationship of proinsulin and insulin with noninsulin-dependent diabetes mellitus and coronary heart disease in Japanese-American men: Impact of obesity-clinical research center study. J. Clin. Endocrinol. Metab..

[49] Despres J.P., Lamarche B., Mauriege P., Cantin B., Dagenais G.R., Moorjani S., Lupien P.J. (1996). Hyperinsulinemia as an independent risk factor for ischemic heart disease. N. Engl. J. Med..

[50] Pyorala M., Miettinen H., Laakso M., Pyorala K. (1998). Hyperinsulinemia and the risk of stroke in healthy middle-aged men: The 22-year follow-up results of the Helsinki Policemen Study. Stroke.

[51] Eschwege E., Richard J.L., Thibult N., Ducimetiere P., Warnet J.M., Claude J.R., Rosselin G.E. (1985). Coronary heart disease mortality in relation with diabetes, blood glucose and plasma insulin levels. The Paris Prospective Study, ten years later. Horm. Metab. Res. Suppl..

[52] Orchard T.J., Eichner J., Kuller L.H., Becker D.J., McCallum L.M., Grandits G.A. (1994). Insulin as a predictor of coronary heart disease: Interaction with apolipoprotein E phenotype. A report from the Multiple Risk Factor Intervention Trial. Ann. Epidemiol..

[53] Becker B., Kronenberg F., Kielstein J.T., Haller H., Morath C., Ritz E., Fliser D., The MMKD Study Group (2005). Renal insulin resistance syndrome, adiponectin and cardiovascular events in patients with kidney disease: The Mild and Moderate Kidney Disease Study. J. Am. Soc. Nephrol..

[54] Shinohara K., Shoji T., Emoto M., Tahara H., Koyama H., Ishimura E., Miki T., Tabata T., Nishizawa Y. (2002). Insulin resistance as an independent predictor of cardiovascular mortality in patients with end-stage renal disease. J. Am. Soc. Nephrol..

[55] Siew E.D., Ikizler T.A. (2008). Determinants of insulin resistance and its effects on protein metabolism in patients with advanced chronic kidney disease. Contrib. Nephrol..

[56] Hung C.J., Huang P.C., Li Y.H., Ho L.T., Chou H.F. (2006). Taiwanese vegetarians have higher insulin sensitivity than omnivores. Br. J. Nutr..

[57] Kuo C.S., Lai N.S., Ho L.T., Lin C.L. (2004). Insulin sensitivity in Chinese ovo-lactovegetarians compared with omnivores. Eur. J. Clin. Nutr..

[58] Scialla J.J., Appel L.J., Wolf M., Yang W., Zhang X., Sozio S.M., Miller E.R., Bazzano L.A., Cuevas M., Glenn M.J. (2012). Plant protein intake is associated with fibroblast growth factor 23 and serum bicarbonate levels in patients with chronic kidney disease: The Chronic Renal Insufficiency Cohort study. J. Ren. Nutr..

[59] Mitch W.E. (2002). Insights into the abnormalities of chronic renal disease attributed to malnutrition. J. Am. Soc. Nephrol..

[60] Trilok G., Draper H.H. (1989). Sources of protein-induced endogenous acid production and excretion by human adults. Calcif. Tissue Int..

[61] Scialla J.J., Anderson C.A.M. (2013). Dietary acid load: A novel nutritional target in chronic kidney disease?. Adv. Chronic Kidney Dis..

[62] Goraya N., Simoni J., Jo C., Wesson D.E. (2012). Dietary acid reduction with fruits and vegetables or bicarbonate attenuates kidney injury in patients with a moderately reduced glomerular filtration rate due to hypertensive nephropathy. Kidney Int..

[63] Key T.J., Fraser G.E., Thorogood M., Appleby P.N., Beral V., Reeves G., Burr M.L., Chang-Claude J., Frentzel-Beyme R., Kuzma J.W. (1999). Mortality in vegetarians and nonvegetarians: Detailed findings from a collaborative analysis of 5 prospective studies. Am. J. Clin. Nutr..

[64] Nanri A.M., Kono S. (2007). Impact of C-reactive protein on disease risk and its relation to dietary factors: Literature review. Asia Pac. J. Cancer Prev..

[65] D’Amico G., Gentile M.G. (1993). Influence of diet on lipid abnormalities in human renal disease. Am. J. Kidney Dis..

[66] Carlsen M.H., Halvorsen B.L., Holte K., Bøhn S.K., Dragland S., Sampson L., Willey C., Senoo H., Umezono Y., Sanada C. (2010). The total antioxidant content of more than 3100 foods, beverages, spices, herbs and supplements used worldwide. Nutr. J..

[67] Appleby P.N., Davey G.K., Key T.J. (2002). Hypertension and blood pressure among meat-eaters, fish-eaters, vegetarians and vegans in EPIC-Oxford. Public Health Nutr..

[68] Mukkuden-Petersen J., Oosthuizen W., Jerling J.C. (2005). A systematic review of the effects of nuts on blood lipid profiles in humans. J. Nutr..

[69] Jenkins D.J., Kendall C.W., Faulkner D., Vidgen E., Trautwein E.A., Parker T.L., Marchie A., Koumbridis G., Lapsley K.G., Josse R.G. (2002). A dietary portfolio approach to cholesterol reduction: Combined effects of plant sterols, vegetable proteins and viscous fibers in hypercholesterolemia. Metabolism.

[70] Liu S., Stampfer M.J., Hu F.B., Giovannucci E., Rimm E., Manson J.E., Hennekens C.H., Willett W.C. (1999). Whole-grain consumption and risk of coronary heart disease: Results from the Nurses’ Health Study. Am. J. Clin. Nutr..

[71] Liu S., Manson J.E., Stampfer M.J., Rexrode K.M., Hu F.B., Rimm E.B., Willett W.C. (2000). Whole grain consumption and risk of ischemic stroke in women: A prospective study. JAMA.

[72] Knekt P., Reunanen A., Jarvinen R., Seppanen R., Heliovaara M., Aromaa A. (1994). Antioxidant vitamin intake and coronary mortality in a longitudinal population study. Am. J. Epidemiol..

[73] Zhou J., Yuan W. (2016). Effects of soy protein containing isoflavones in patients with chronic kidney disease: A systematic review and meta-analysis. Clin. Nutr..

[74] Zhang J., Liu J., Su J., Tian F. (2014). The effects of soy protein on chronic kidney disease: A meta-analysis of randomized controlled trials. Eur. J. Clin. Nutr..

[75] Kandouz S., Mohamed A.S., Zheng Y., Sandeman S., Davenport A. (2016). Reduced protein bound uraemic toxins in vegetarian kidney failure patients treated by haemodiafiltration. Hemodial. Int..

[76] Wu T.T., Chang C.Y., Hsu W.M., Wang I.-K., Hsu C.-H., Cheng S.-H., Liang C.-C., Chang C.-T., Huang C.-C. (2011). Nutritional status of vegetarians on maintenance haemodialysis. Nephrology (Carlton).

[77] Piccoli G., Attini R., Vesario E., Gaglioti P., Piccoli E., Consiglio V., Deagostini C., Oberto M., Todros T. (2011). Vegetarian supplemented low-protein diets. A safe option for pregnant CKD patients: Report of 12 pregnancies in 11 patients. Nephrol. Dial. Transplant..

[78] Attini R., Leone F., Parisi S., Fassio F., Capizzi I., Loi V., Colla L., Rossetti M., Gerbino M., Maxia S. (2016). Vegan-vegetarian low-protein supplemented diets in pregnant CKD patients: Fifteen years of experience. BMC Nephrol..

[79] Cahill L.E., Peng C.Y., Bankovic-Calic N., Sankaran D., Ogborn M.R., Aukema H.M. (2007). Dietary soya protein during pregnancy and lactation in rats with hereditary kidney disease attenuates disease progression in offspring. Br. J. Nutr..

[80] Bonacasa B., Siow R.C., Mann G.E. (2011). Impact of dietary soy isoflavones in pregnancy on fetal programming of endothelial function in offspring. Microcirculation.

[81] Chauveau P., Couzi L., Vendrely B., de Precigout V., Combe C., Fouque D., Aparicio M. (2009). Long-term outcome on renal replacement therapy in patients who previously received a keto acid-supplemented very-low-protein diet. Am. J. Clin. Nutr..

[82] Garneata L., Stancu A., Dragomir D., Stefan G., Mircescu G. (2016). Ketoanalogue-Supplemented Vegetarian Very Low-Protein Diet and CKD Progression. J. Am. Soc. Nephrol..

